# Wasp Hawking Induces Endothermic Heat Production in Guard Bees

**DOI:** 10.1673/031.010.14102

**Published:** 2010-09-09

**Authors:** K. Tan, H. Li, M.X. Yang, H.R. Hepburn, S.E. Radloff

**Affiliations:** ^1^ Xishuangbanna Tropical Botanical Garden, Chinese Academy of Science, Kunming, Yunnan Province, 650223, People's Republic of China; ^2^ Eastern Bee Research Institute of Yunnan, Agricultural University, Heilongtan, Kunming, Yunnan Province, People's Republic of China; ^3^ Department of Zoology and Entomology, Rhodes University, Grahamstown 6140, South Africa; ^4^ Department of Statistics, Rhodes University, Grahamstown 6140, South Africa

**Keywords:** *Apis cerana*, *Apis mellifera*, *Vespa velutina*, temperature

## Abstract

When vespine wasps, *Vespa velutina* Lepeletier (Hymenoptera: Vespidae), hawk (capture) bees at their nest entrances alerted and poised guards of *Apis cerana cerana* F. and *Apis mellifera ligustica* Spinola (Hymenoptera: Apidae) have average thoracic temperatures slightly above 24° C. Many additional worker bees of *A. cerana,* but not *A. mellifera*, are recruited to augment the guard bee cohort and begin wing-shimmering and body-rocking, and the average thoracic temperature rises to 29.8 ± 1.6° C. If the wasps persist hawking, about 30 guard bees of *A. cerana* that have raised their thoracic temperatures to 31.4 ± 0.9° C strike out at a wasp and form a ball around it. Within about three minutes the core temperature of the heat-balling *A. cerana* guard bees reaches about 46° C, which is above the lethal limit of the wasps, which are therefore killed. Although guard bees of *A. mellifera* do not exhibit the serial behavioural and physiological changes of *A. cerana*, they may also heat-ball hawking wasps. Here, the differences in the sequence of changes in the behaviour and temperature during “resting” and “heat-balling” by *A. cerana* and *A. mellifera* are reported.

## Introduction

*Vespa velutina* Lepeletier (Hymenoptera: Vespidae), a vespine wasp endemic to southeast Asia, preys on honeybees, both the native *Apis cerana cerana* F. (Hymenoptera: Apidae) as well as the introduced European *Apis mellifera* (Hymenoptera: Apidae) (Matsuura and Yamane 1990; Tan et al. 2005; Tan et al. 2007). The wasps hawk (capture) foraging honeybees on the wing near honeybee colonies, and predation is especially fierce in autumn when *V. velutina* are most populous (Li 1993). While native *A. cerana* colonies have evolved defense strategies against *V. velutina* predation, the introduced *A. mellifera* sustains significantly greater losses than the former (Qun 2001; Tan et al. 2005; Tan et al. 2007). If *V. velutina* come close to a honeybee nest, the guard bee cohort increases, shimmers their wings, and, if *V. velutina* persist, the guard bees launch strikes to kill them by heat-balling (Tan et al. 2005; Tan et al. 2007).

Such endothermic heat is generated by the thoracic musculature (Esch 1960; Esch and Goller 1991; Stabentheiner et al. 2003; Kunieda et al. 2006) and facilitates pre-flight warm up (Krogh and Zeuthen 1941; Heinrich 1979; Esch et al. 1991), brood incubation (Bujok et al. 2002), heat balling (Ono et al. 1987; Tan et al. 2005; Tan et al. 2007), and other defensive contexts (Southwick and Moritz 1985). When many worker bees ball a wasp, they can kill it by raising the ball core temperature to 46° C in about 3 min (Matsuura and Sakagami 1973; Ono et al. 1987; Tan et al. 2005). However, the “resting” temperatures of guard bees at a hive entrance are considerably lower than those reached during heat balling. Because a “resting” guard bee has a temperature of about 24° C, which nearly doubles to 46° C during heat balling, the hypothesis for this study was that such a physiological thermal jump must be graded and ought to be reflected in more gradual changes in the transition from simply guarding to active heat balling. The sequence of changes in the behaviour and thoracic temperatures of guard bees was examined during the transition from “resting” to poised alert, to wing-shimmering, and finally, to heat-balling attacks by *A. cerana* and *A. mellifera* against *V. velutina* hawking at the hive entrance.

## Materials and Methods

Six colonies (three *A. cerana* and three *A. mellifera*) of equal size, four combs of about 10,000 bees each, were tested in autumn (September–October 2008) in an apiary at Yunnan Agricultural University, Kunming, China. Because subspecies of *A. mellifera* differ greatly in defensive behaviour (Hepburn and Radloff 1998), the Italian bee, *A. mellifera ligustica* Spinola, was used. This is the principal *mellifera* race that is used commercially in China.

In the bioassays, a live *V. velutina* wasp was suspended from a horizontally placed wire by a piece of cotton tied around its petiole. The wasp was held about 20 cm away from the entrance of a hive and could fly and move freely within the confines of the length of the cotton. Its movements would alert the guard bees. For each bee colony, the thoracic temperatures of 20 individual guard bees were measured in the absence of *V. velutina* as the control group, and 20 more bees were measured after presentation of the live wasps as the test group in experiment 1. In a second experiment, dead, dichloromethane-extracted, washed, and dried *V. velutina* served as the controls against live wasps.

The body temperatures of the guard bees were measured about 20–30 cm away from the entrance of the hive with a hand-held laser infrared digital thermometer with a resolution of ± 0.1° C (AZ^@^ Model 8889, AZ Instrument Corp, www.az-instrument.com.tw). During the tests, ambient temperature was about 2123° C. A digital video camera (Panasonic NVGS400GC, www.panasonic.com) was placed 1 m in front of the hive entrances to record the shimmering and wasp-striking behaviour of the guard bees during the thermomeasurement. Just when the guard bees were launching to strike the wasp, their instantaneous thoracic temperatures were immediately measured in an area of about 4 mm^2^. For each colony, 10 individual striking bees and 10 guard bees that did not strike were measured.

## Results

**Figure 1.**
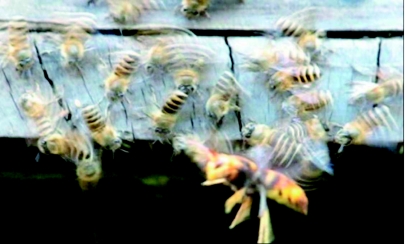
The wing shimmering behaviour of *Apis cerana* guard bees when a live vespine wasp, *Vespa velutina*, is placed at the entrance of an *A. cerana* hive. High quality figures and videos are available online.

When live wasps were placed at the entrance of an *A. cerana* hive, changes in the thoracic temperature of guard bees increased significantly from a resting temperature of 24.3 ± 1.1° C to 29.8 ± 1.6° C during shimmering (Dependent *t*-test, t_59_ = 21.9, p < 0.001, Figure 1, Video). However, exposure to wasps had no significant effect on the thoracic temperature of *A. mellifera* guard bees for the same test (Dependent *t*-test, t_59_ = 0.24, p = 0.81, Table 1). There were significant differences for both the control and test groups between *A. cerana* and *A. mellifera* (Independent *t*-test, Control: t_118_ = 2.6, p = 0.01; Test: t_118_ = 18.1, p < 0.001; Table.1). When *V. velutina* flies near *A. cerana* guard bees, some attack and engulf it in the core of the heat balling bees. The thoracic temperatures of guard bees just on the verge of a strike increased by 1.7 ± 1.8° C to 31.4 ± 0.9° C, which is significantly higher than that of alert but non-striking guard bees (29.7 ± 1.6°C) (Independent t-test, t_58_ = 5.0, p < 0.001).

When dead *V. velutina* wasps were presented to *A. cerana* and *A. mellifera* in the second test, there was no significant difference in the mean thoracic temperatures of guard bees between the control group with no dead wasp and the test group with a dead *V. velutina* for both *A. cerana* (t_118_ = 0.04, p = 0.97) and *A. mellifera* (t_188_ = 1.6, p = 0.11).

## Discussion

Although heat-balling wasps as such is well documented (Ono et al. 1987; Tan et al. 2005), the behavioural sequence of attracting additional recruits to the guard bee cohort, increased numbers of wing-shimmering guard bees (an average of 32.2 ± 3.2 bees/ball (Tan et al. 2005)) that raise thoracic temperature prior to striking *V. velutina* have not been previously measured for either *A. cerana* or *A. mellifera.* Un-alerted guard bees of both *A. cerana* and *A. mellifera* have relatively low thoracic temperatures, about 24° C, but when hawking *V. velutina* approach them, unlike *A. mellifera, A. cerana* guard bees are immediately alerted and begin body shaking and wing shimmering. Likewise, thoracic temperature rapidly increases some 5.4 ± 1.9° C, and those guard bees with the higher thoracic temperatures more readily attack *V. velutina* than those at lower temperature. The wing shimmering behaviour is directly associated with increasing the guard bee cohort, and may be mediated by the simultaneous release of a pheromone. Because shimmering guard bees increase their surface temperatures during wing-shimmering, this would facilitate the dispersal of any recruiting pheromones (Stabentheiner et al. 2002). Likewise, during fanning *A. cerana* face away from the nest entrance (Sakagami 1960), and this would direct any pheromonal plume backwards into the nest. However, it has been reported that *A. cerana* does not expose its Nasanov gland during shimmering (Koeniger et al. 1996). Wing-shimmering is also interpreted as an anti-predator visual pattern disruption mechanism, similar to that of *A. nuluensis* (Koeniger et al. 1996).

**Video.**
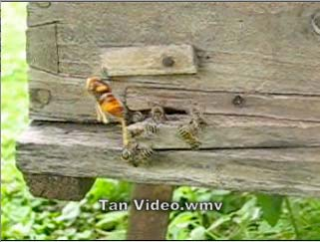
Response of *Apis cerana* guard bees to a vespine wasp, *Vespa velutina,* flying near the entrance of the *A. cerana* hive. High quality figures and videos are available online.

**Table 1.**
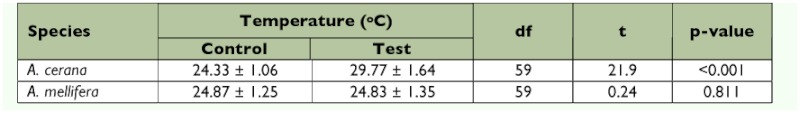
Means and standard deviations for body temperatures (°C) of *Apis cerana* and *Apis mellifera* guard bees before and after the appearance of wasps (dependent samples t-test)

In contrast, *A. mellifera* guard bees do not exhibit these behavioural responses to hawking *V. velutina,* and there is no rapid elevation of thoracic temperature. This apparent inability to rapidly detect *V. velutina* and to respond defensively accounts for the greater *V. velutina* presence and hawking success rate at colonies of *A. mellifera* than *A. cerana* in autumn (Tan et al. 2007). Moreover, *A. cerana* may also withdraw into its nest or use wing-shimmering, traits absent from the behavioural repertoire of *A. mellifera.* However, wasp-balling behaviour is exhibited by *Apis mellifera cypria,* which apparently kills wasps by asphyxiation (Papachristoforou et al. 2007).

In any event, *V. velutina* preferentially hawk *A. mellifera* foragers when both *A. mellifera* and *A. cerana* occur in the same apiary (Tan et al. 2007). The present observations suggest a reciprocal co-evolution in the prey/predator relationship between *V. velutina* and *A. cerana,* both of which are endemic to and sympatric in southeast Asia (Li 1993; Tan et al. 2005), while *A. mellifera* was introduced from Europe, where there is no widespread *V. velutina* predation. The fact that the behavioural sequences described here for *A. cerana* also occur in *Apis nuluensis* (Koeniger at al. 1996) and *Apis dorsata* (Kastberger et al. 1998; Kastberger and Stachl 2003) suggests that such anti-predator behavioural adaptations may be widespread between predators and honeybees in southeast Asia.
